# Current News and Opinion

**Published:** 1882-10

**Authors:** 


### CURRENT NEWS AND OPINION.

The London Medical Schools opened October 2d ; the period during which students could enter for the winter closing on the 15th.

It has just been reported that only nine states are now without a State Board of Health, and that in most of these steps are being taken to form a Board. The delinquent States are as follows : Florida, Kansas, Maine, Missouri, Nebraska, Nevada, Ohio, Pennsylvania and Vermont.

The Baltimore Academy of Medicine has recently been incorporated.

Chiari, of Vienna, has been called to the chair of Pathological Anatomy at Prague, as successor of Klebs.

Dr. Ezra B. Bennett, who died at Danbury, Conn., on Oct. 27th, was one of the oldest physicians in that state, being an active practitioner for over fifty-three years. His special interest was early called to surgery, and he was the pioneer in operating for ovarian tumor, visigo, vaginal fistula and club-foot.

The number of sane persons who hava been imprisoned at Ward's Island Asylum, through malice or other causes, has been the means of bringing the matter before the Grand Jury, who have commenced an investigation of the management.

An organization called the Union of Women of France, having for its object the caring for wounded in time of war and of those who have been injured by public disasters, in its annual report spoke of the services rendered by this Society at Algiers, Tunis, Boulogne, and other places. Practical instruction has been offered to pupils of the Union, which will make trained and experienced nurses.

Mr. Geo. E. Waring Jr. has been appointed member of the National Board of Health, vice Dr. Chas. F. Folsom, resigned.

It has often been a question of doubt as to the base and infamous poison which was used by the Borgias. Perret makes the assertion that it was the oil of ergot.

Smallpox is raging in Cincinnati. The Cincinnati *Lancet and Clinic* in a lengthy editorial considers the disease and its treatment.

The *Sanitary Engineer* is now publishing the reports of the U. S. Board of Health.

